# Preparation of Hydrogarnet/Poly(Lactic Acid) Composite Adsorbents for Humic Substance Removal

**DOI:** 10.3390/ma16010336

**Published:** 2022-12-29

**Authors:** Shogo Minowa, Hirotaka Maeda

**Affiliations:** Department of Life Science and Applied Chemistry, Nagoya Institute of Technology, Gokiso-cho, Showa-ku, Nagoya 466-8555, Japan

**Keywords:** adsorbent, hydrogarnet, poly (lactic acid), humic acid removal, porous structure

## Abstract

Humic substances are constituents of organic matter that require removal from water environments because of their adverse ecological and sanitation effects. A mixture of hydrogarnet and poly(lactic acid) dissolved in chloroform was electrospun to prepare a composite as a adsorbent for humic substance removal. Here, humic acid was used as the model substance for evaluating the adsorbent’s water remediation efficiency. Despite the hydrogarnet particles being embedded in its poly(lactic acid) fibers, the composites demonstrated a higher humic acid removal ability than the pure poly(lactic acid) sample prepared using an electrospinning process. Pores were introduced to the fiber surfaces of the composite by controlling the relative humidity during electrospinning, thus enhancing their humic acid removal ability (4.6 ± 2.4 mg/g), compared to the composite consisting of the fibers without pores (1.2 ± 0.9 mg/g).

## 1. Introduction

Natural organic matter formed by the decomposition of animals and biomass are a pollutant of concern in aqueous environments. Humic substances, classified as humic acid (HA), fulvic acid, and humin, are the major constituents of organic matter. As these substances can cause colorization in water, and their presence has a negative effect on the adsorption properties of other compounds [1], they should be removed from aqueous environments. However, humic substances tend to react with oxidants used in water purification to form carcinogenic byproducts such as trihalomethanes [2]. Hence, principles such as adsorption, coagulation, and photocatalysis have been suggested for the removal of humic substances from water [3,4], with adsorption processes demonstrating economic benefits.

Materials such as activated carbon and zeolite have been investigated as adsorbents for use in humic substance removal [5,6]. More recently, we found that hydrogarnet powders prepared from a CaO-SiO_2_-Al_2_O_3_-H_2_O system using a hydrothermal process are excellent adsorbers of HA. This is because of the tuning the chemical compositions of presence of Ca_3_Al_2_(OH)_12_ and Ca_3_Al_2_(SiO_4_)_3_ in hydrogarnets (Ca_3_Al_2_(SiO_4_)_3−x_(OH)_4x_, with x = 0–3), which gives them a stronger affinity for HA than zeolite and activated carbon [7]. It has been reported that Ca_3_Al_2_(OH)_12_ exhibited phosphate adsorption performance in aqueous solution [8]. Remediation processes that rely on hydrogarnets containing the SiO_4_ units thus offer the potential for more effective humic substance removal.

In addition to its chemical composition, the morphology of an adsorbent determines its ability to handle water remediation processes. Although fabrics with porous structures have been proven to be a suitable shape for humic substance removal, creating such fabrics consisting of only hydrogarnets is difficult; membranes are typically created using ceramic processes, which can alter the structure of the hydrogarnet, (e.g., by degrading hydroxyl groups) because of the high temperatures they require. Porous polymer membranes are one of candidate materials for water remediation applications [9,10]. An approach to resolve this problem is to embed partially hydrogarnet particles into a porous polymer matrix. Hence, this study discusses the preparation of composite adsorbents containing hydrogarnet, for humic substance removal.

Porous polymer matrix can be created from non-woven fiber fabrics using electrospinning and spun bonding processes. Many reports have been published on the preparation of ceramic/polymer composite adsorbents using electrospinning [11,12], suggesting that this technique is suitable for combination with hydrogarnet. Furthermore, in general, the fiber diameters of fabrics prepared using an electrospinning process are finer than the diameters of fabrics prepared using a spun bonding process. We thus surmise that fabrics created using an electrospinning method are more suitable for water remediation because of their relatively high contact area with target substances, resulting from the fine fiber diameters. Hence, in this study, the composite adsorbents containing hydrogarnet were prepared using an electrospinning technique. Here, poly-L-lactic acid (PLLA) was used as the polymer phase because it is biodegradable, and more stable in water environments than other degradable polymers. Humic acid was used as the model humic substance for determining the composite adsorbents effectiveness in water remediation. Finally, all water remediation tests were also performed with a pure polymer adsorbent, to distinguish the effect of membrane structure on humic substance removal from the effect of hydrogarnet adsorption.

## 2. Materials and Methods

### 2.1. Preparation of the Samples

**Figure 1:**
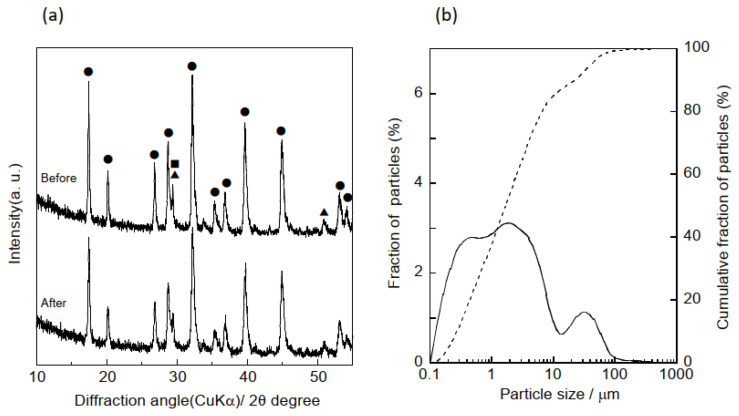
(**a**) XRD patterns of hydrogarnet before and after the ball milling (●) hydrogarnet, (▲) C-S-H gel, (■) calcite, and (**b**) particle size distribution curves of hydrogarnet after the ball milling. Solid and dotted lines indicates the fraction and cumulative fraction of particle size, respectively.

To prepare the hydrogarnet using our previously reported method [13], calcium hydroxide (Ca(OH)_2_, Fujifilm Wako Pure Chemical Corp., Osaka, Japan), γ-alumina (Al_2_O_3_, Taimei Chemicals Co., Ltd., Kamiina-gun, Nagano, Japan), and silica gel (SiO_2_, Fuji Silysia Chemical Ltd., Kasugai, Japan) powder were mixed in a 3:2:1 molar ratio of Ca:Al:Si. Distilled water was added to this mixture to prepare a slurry with a 1:5 solid-to-liquid ratio. The slurry was stirred and hydrothermally treated at 150 °C for 8 h. Following treatment, the slurry was filtered, and the resulting solids were dried at 60 °C for 12 h. The solids were pulverized to form a powder by ball milling at 400 rpm for 10 min in methanol. The average particle size (D_50_) of the pulverized powder was determined to be approximately 1.7 µm using a laser diffractometer, as shown in Figure 1a. In addition, XRD of the powder demonstrated that ball milling had no influence on the crystalline phase of the hydrogarnet (Figure 1b).

Crystalline PLLA (170 kDa) obtained from Mitsui Chemicals, Inc., LACEA was dissolved in chloroform (Wako Pure Chemical Industries, Ltd., Richmond, VA, USA) to a concentration of 12 mass%. Here, chloroform was selected as the solvent because of its favorable evaporation properties [14]. The pulverized powder was added to the PLLA solution in a 1:4 mass ratio of hydrogarnet to PLLA, and the mixture was stirred at 50 rpm for 24 h. It is well known that non-woven fiber fabrics can be obtained by controlling the solution and process parameter. To ensure the composite samples were reproducible, the optimal solid/liquid ratio for the hydrogarnet/PLLA mixture used in the electrospinning process was determined based on a trial-and-error approach. The solid/liquid ratio of the resultant slurry was 3/17. The mixture was poured into a glass syringe with an 18 G stainless steel needle. Electrospinning was performed by applying a 20 kV voltage to the needle. The mixture was extruded from the needle for 210 s at a feed rate of 0.05 mL/h. The fibers were collected on a grounded plate covered with aluminum foil located 15 cm from the needle. Electrospinning was conducted at a relative humidity of approximately 20%, regulated using silica gel. The pure polymer sample was prepared from a PLLA solution without additives using the same procedure.

### 2.2. Characterization of the Samples

The morphologies and electron mappings of the samples were observed using scanning electron microscopy (SEM) incorporating an energy-dispersive spectrometer (EDS) with an accelerating voltage of 5 or 10 kV. The crystal phases of the samples were analyzed using X-ray diffraction (XRD) in the range of 20° to 45° with 0.015 deg/s of the scanning rate. The fiber diameter of a sample was calculated from the average of at least 50 fibers using ten SEM images. The samples were analyzed via attenuated total reflectance (ATR)-Fourier transform infrared (FT-IR). The static contact angle immediately after the water droplet (1 μL) attached to the surface of the samples was measured using a contact angle analyzer.

### 2.3. Analysis of the Water Remediation Ability of the Samples

**Figure 2:**
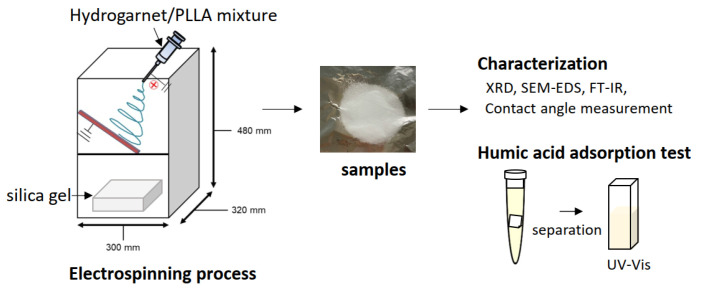
Experimental scheme of this study.

HA adsorption tests were performed batchwise, by soaking a 10 mm × 10 mm sample in 10 mL of a HA solution at pH = 7 adjusted by 0.1 mol/L NaOH and HCl with an initial concentration of 5 ppm, which was the similar value to the HA concentration of spring water in the Tsuruma park (Nagoya, Aichi). HA was not adsorbed on all samples after 6 h of immersion. This solution was stirred at room temperature for 24 h during the adsorption tests. Following immersion, the test solution at least four different specimens was analyzed using a UV-Vis spectrometer, by monitoring the change in absorbance at 250 nm. HA in solution was proportional to color measured at around 250, 270 and 410 nm absorbance [15]. In each adsorption test, the absorbance of a blank cell without a sample was measured as a reference. The HA concentration of the resultant solution was determined from the linear equation prepared by plotting the absorbance at 250 nm against the HA concentration in the in the range of 0–5 ppm. The uncertainly was evaluated as the standard deviation of the measurements. The experimental scheme is shown in Figure 2.

## 3. Results

### 3.1. Characterization of Samples

**Figure 3:**
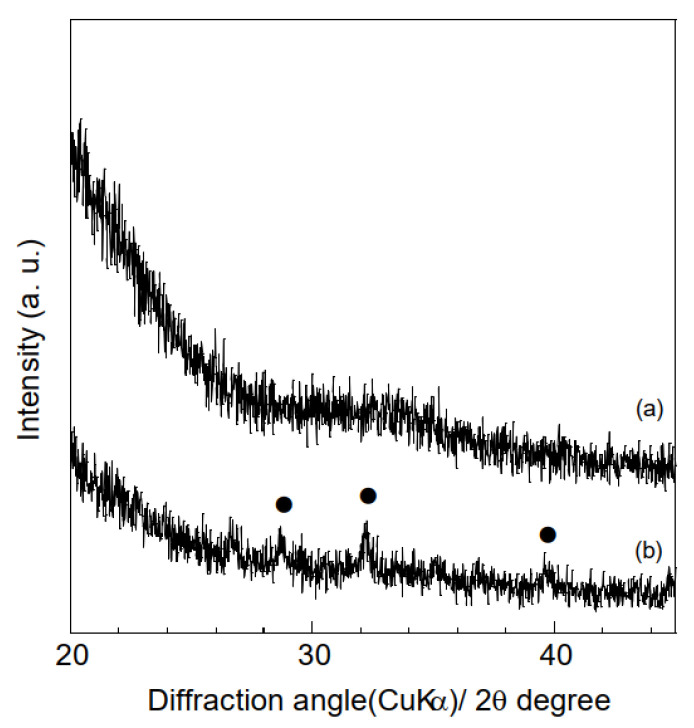
XRD patterns of the (**a**) polymer and (**b**) composite samples. (●) Hydrogarnet.

**Figure 4:**
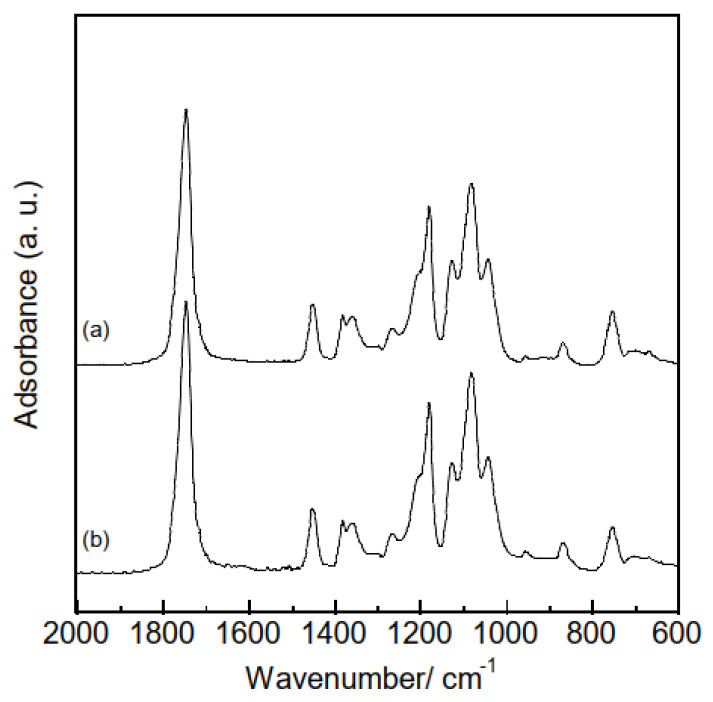
ATR-FT-IR spectra of the (**a**) polymer and (**b**) composite samples.

A range of characterization analyses were conducted on the polymer and composite samples prior to HA adsorption tests, to verify successful electrospinning. The thicknesses of the samples were evaluated based on the averages and standard deviations of ten measurements obtained using a micrometer. The thicknesses of the polymer and composite samples were 154 ± 24 μm and 118 ± 31 μm, respectively, indicating that combining hydrogarnets with PLLA has little effects on the thickness. Figure 3 shows the XRD patterns of the samples. Although crystalline PLLA was used as the starting material, none of the samples generated diffraction peaks at angles below 20°. This indicates that following electrospinning, the PLLA in the samples exists in the amorphous phase, which agrees with the literature [16]. Peaks corresponding to hydrogarnet were observed in the XRD pattern of the composite samples, confirming its successful combination with PLLA. There were almost the same ATR-FT-IR spectra in the polymer and composite samples, as shown in Figure 4. This implies that chemical bonds of PLLA would have no change even after adding hydrogarnet. Thermogravimetric analysis conducted in our previous work clarified that following thermal decomposition of PLLA fiber membranes, weight loss occurred between 300 °C and 400 °C [17]. In this study, the hydrogarnet content of the composite sample was estimated to be 12.0 mass% based on the weight of the residue obtained after heat treatment at 450 °C. This value is approximately half of the preparation composition, suggesting that the hydrogarnet powder was not dispersed homogeneously in the PLLA mixture.

**Figure 5:**
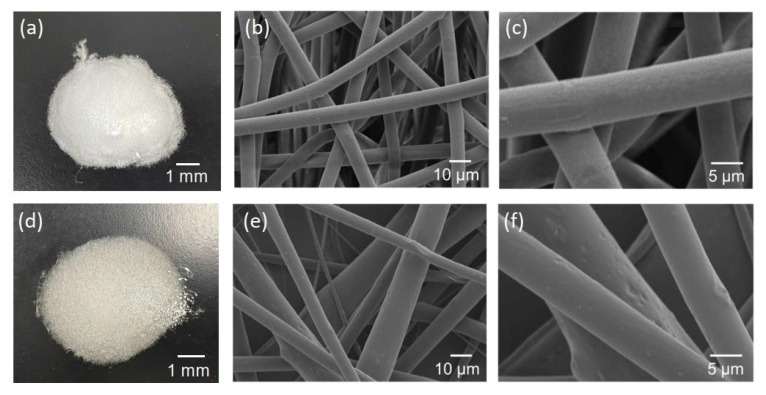
Pictures (left column) and SEM micrographs (middle and right columns) of the (**a**–**c**) polymer and (**d**–**f**) composite samples.

**Figure 6:**
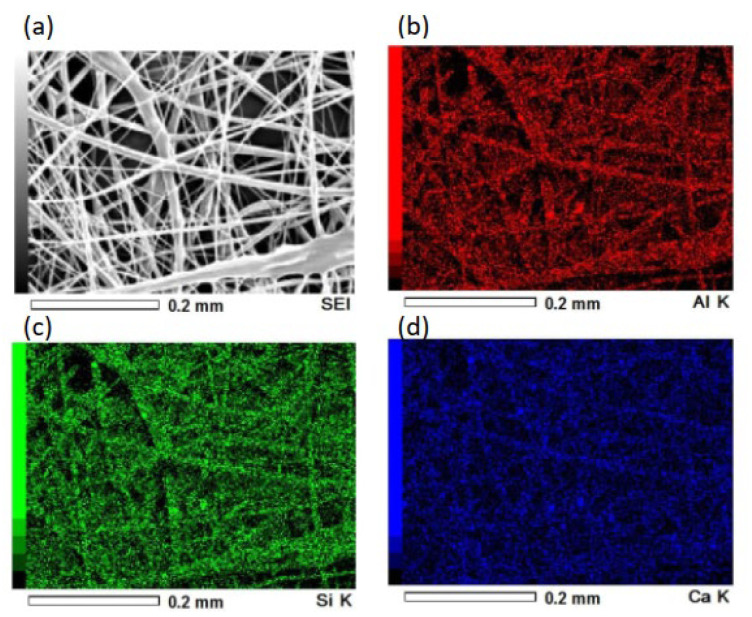
(**a**) Secondary electron image and EDS mapping image for (**b**) Al, (**c**) Si and (**d**) Ca of the composite sample.

**Figure 7:**
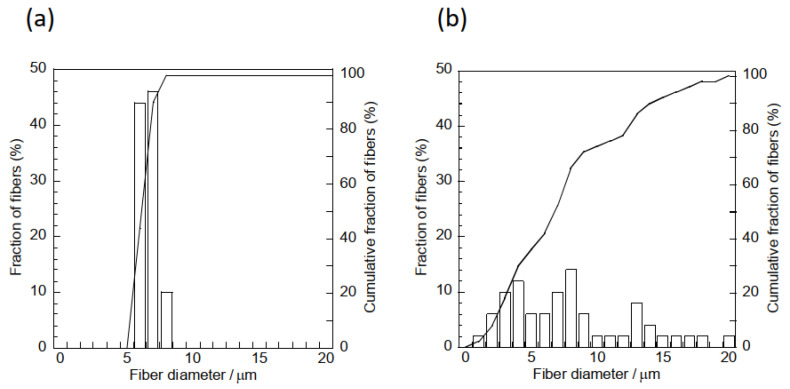
Fiber size distribution curves of the (**a**) polymer and (**b**) composite samples.

Pictures and SEM micrographs of the samples are shown in Figure 5. The SEM images confirm that each sample consists of micrometer-sized fibers. There was absence of particles on the surfaces of the fibers of the composite sample. Al, Ca and Si elements, which are components of hydrogarnet, were detected like overlapped with the fibers by EDS analysis, as shown in Figure 6. These indicate that the hydrogarnet is embedded within the fibers. Fine fibers can also be observed with the composite sample, which were not observed in the polymer sample. The distribution of the fiber diameters estimated from the SEM images are shown in Figure 7. This figure indicates that the polymer sample consists of a more monodisperse population of fibers, with diameters ranging from 6 µm to 8 µm. In contrast, the diameters of the fibers in the composite sample ranged from 1 µm to 20 µm. It has been reported that the viscosity and surface tension of the polymer solution used in electrospinning affect fiber diameter [18,19]. The introduction of hydrogarnet powder to the PLLA solution affects these properties, resulting in the heterogeneity of the fiber diameters in the composite sample.

### 3.2. Humic Acid Adsorption Proerty of the Samples

The polymer sample did not adsorb any HA. A 1.2 ± 0.9 mg/g concentration of HA was adsorbed onto the composite samples after 24 h of immersion. Spherical activated carbon for liquid phase (As Ones Corp.) used as the comparison material, provided 1.1 mg/g of HA adsorption by soaking a 10 mg in 10 mL of a HA solution with an initial concentration of 5 ppm. This means that the composite sample has equivalent potential to HA adsorption properties of materials used as a adsorbent. The polymer and composite samples indicated their contact angles of 128.8 ± 1.4° and 126.1 ± 1.2°, respectively. The introduction of hydrogarnet into the PLLA matrix has no influence on the contact angles. Our preliminary experiments indicated that a composite sample prepared using a mixture with 1:5 mass ratio of HG:PLLA did not adsorb HA even after 24 h of immersion. This implies that the hydrogarnet content of a composite sample must exceed a minimum amount for it to be able to remove HA from effluent.

**Figure 8:**
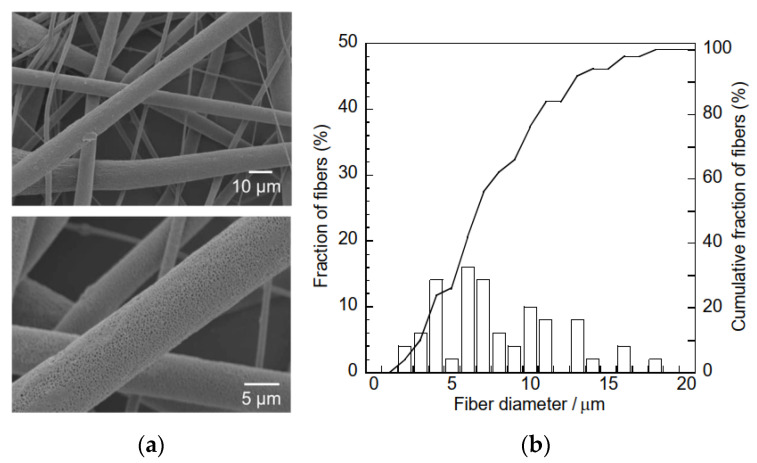
(**a**) SEM micrographs and (**b**) fiber size distribution curves of the composite sample consisting of porous fibers.

**Figure 9:**
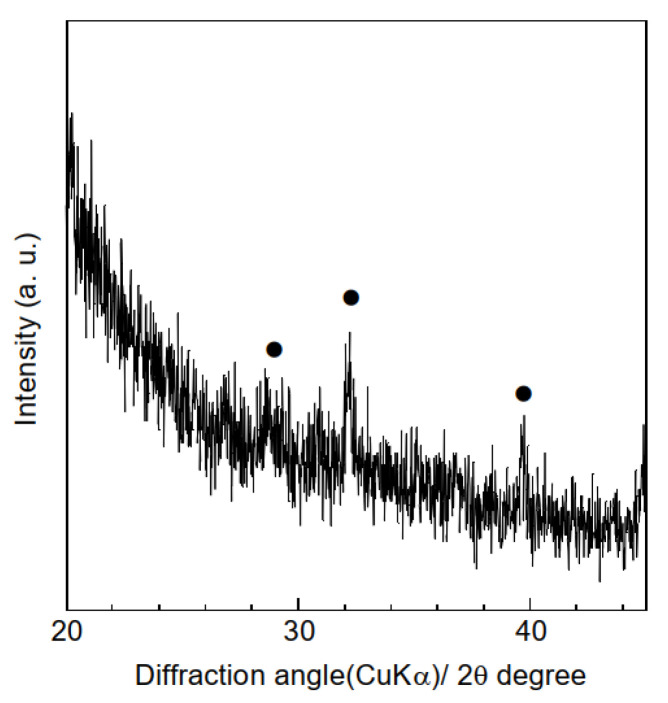
XRD patterns of the composite sample consisting of porous fibers. (●) Hydrogarnet.

To improve the HA adsorption capacity of the composite sample, the hydrogarnet content of the precursor polymer mixture can be increased. However, this increased hydrogarnet content increases the polymer solution’s viscosity, leading to electrospinning failure. An alternative solution is to construct a composite sample with porous fibers, as the inclusion of pores enables easier penetration of effluent into the fibers, and subsequent contact with the hydrogarnet particles. It has been reported that performing electrospinning in a relatively high humidity environment generates pores in the fibers of the resulting samples [20,21]. Hence, in this study, to improve the composite sample’s HA adsorption, the relative humidity during the electrospinning process was regulated to approximately 75% using an NaCl-H_2_O mixture. This increased humidity had minimal effect on the thickness of the resulting sample (112 ± 16 μm) and its fiber diameter distribution, as shown in Figure 8. Similarly, peaks corresponding to hydrogarnet were observed in the corresponding XRD pattern (Figure 9) indicating that the humidity did not affect the combination of hydrogarnet with PLLA. However, numerous pores appeared on the surfaces of the fibers. The hydrogarnet content in the composite sample consisting of porous fibers was estimated to be 9.0 mass%, which is similar to that of the sample consisting of non-porous fibers. The contact angles of the composite sample consisting of porous fibers were determined to be 129.4 ± 1.9°, which is almost the same as that with non-porous fibers, indicating that both samples have similar contact areas with HA. However, the composite sample consisting of porous fibers adsorbed 4.6 ± 2.4 mg/g of HA after 24 h of immersion, as shown in Table 1. This value is much higher than the HA concentration adsorbed by the composite sample with non-porous fibers. Given their similar hydrogarnet content and contact angle, this result indicates that the existence of pores on a fiber surface enhances the HA adsorption capacity of a composite sample by enhancing the ability of HA to penetrate inside the fibers and react with hydrogarnet. In addition, the composite samples consisting of porous fibers maintained their shape even after humic acid removal tests. The thermally activated coal-based carbons with and without acid wash treatment showed around 3–20 mg/g of the amount adsorbed HA at equilibrium at pH = 7, dependently of a function of the nonadsorbed HA per unit carbon mass [22]. The maximum sorption capacity of goethite, which was commercially available, was reported to 3.8–5.6 mg/g at different pHs [23]. The composite sample consisting of porous fibers seems to have a similar potential as the previous reported adsorbents for HA removal. Recently, we have successfully improved the HA adsorption properties of hydrogarnet by heat treatment [24]. Introduction of the treated hydrogarnet into the composite would enhance the HA adsorption properties. The pH of the HA solution and dose of the adsorbents influence their adsorption properties [25]. Investigation with respect to the effect of the pH, the adsorbent dose and its reusable performance are in progress to discuss the HA adsorption properties of the composite sample more in-depth.

## 4. Conclusions

This study demonstrated the creation of composite hydrogarnet/poly(lactic acid) adsorbents for humic substance removal using an electrospinning technique. An combination of hydrogarnet with electrospun PLLA sample was required for the adsorbents to demonstrate humic acid removal. Controlling the humidity during electrospinning introduced pores on the fiber surfaces of the samples, which enhanced their humic acid removal. This study suggested that hydrogarnet/PLLA composite consisting of porous fibers, which was similar potential as the previous reported adsorbents, is a promising adsorbent for HA removal.

## Figures and Tables

**Table 1:** Humic acid adsorption properties of the samples.

Polymer Sample	Composite Sample Consisting of Non-Porous Fibers	Composite Sample Consisting of Porous Fibers
No adsorption	1.2 ± 0.9 mg/g	4.6 ± 2.4 mg/g

## Data Availability

The data presented in this study are available on request from the corresponding author.
